# Using a Back‐Worn Accelerometer to Capture Nocturia Frequency in Parkinson's: An Exploratory Study

**DOI:** 10.1002/nau.70011

**Published:** 2025-02-17

**Authors:** Anisha Cullen, Matthew D. Smith, Emily J. Henderson

**Affiliations:** ^1^ Population Health Sciences, Bristol Medical School University of Bristol Bristol UK; ^2^ Department of Neurology North Bristol NHS Trust Bristol UK; ^3^ Older Peoples Unit Royal United Hospital NHS Foundation Trust Bath UK

## Clinical Trial Registration

The STRIPE trial was registered on 22 June 2021 (ISRCTN11484954). Additional registration was not required for this sub study.

Parkinson's disease (PD) affects one in 37 people in their lifetime and is the second most common neurodegenerative disorder [1]. PD not only affects mobility but presents with multiple non‐motor symptoms, with lower urinary tract symptoms (LUTS) reported to affect around two‐thirds [2]. These have a negative impact on quality of life [3], and have been identified as a research priority by people with PD [4]. Nocturia, defined as a need to void urine during the main sleep period [5], is frequently reported as the most common LUTS in PD, with prevalence ranging from 60% [6] to 86% [7].

Currently, bladder diaries are the gold standard for capturing nocturia and quantifying episodes [8]. However, they are subject to recall bias, omissions and legibility difficulties; heightened in a PD population where cognitive impairment and issues with dexterity and mobility are prevalent. Accelerometers are frequently used to capture movement in real‐world settings and body worn electronic devices are unobtrusive and offer the potential to capture movement objectively. However, limited studies have used accelerometers to capture urination [9], with studies conducted in animals [10] or focusing on stress urinary incontinence [11]. We conducted an exploratory study to determine whether accelerometers could accurately capture nocturia compared to bladder‐diaries.

This was a sub‐study of the STRIPE randomized control trial of tibial nerve stimulation in PD described elsewhere [12]. Participants were recruited based on a clinical history of overactive bladder, and completed a 72‐h bladder‐diary at baseline (pre‐intervention) and the study end (post‐intervention), a number with nocturia were invited to trial the accelerometer.

**Figure 1:**
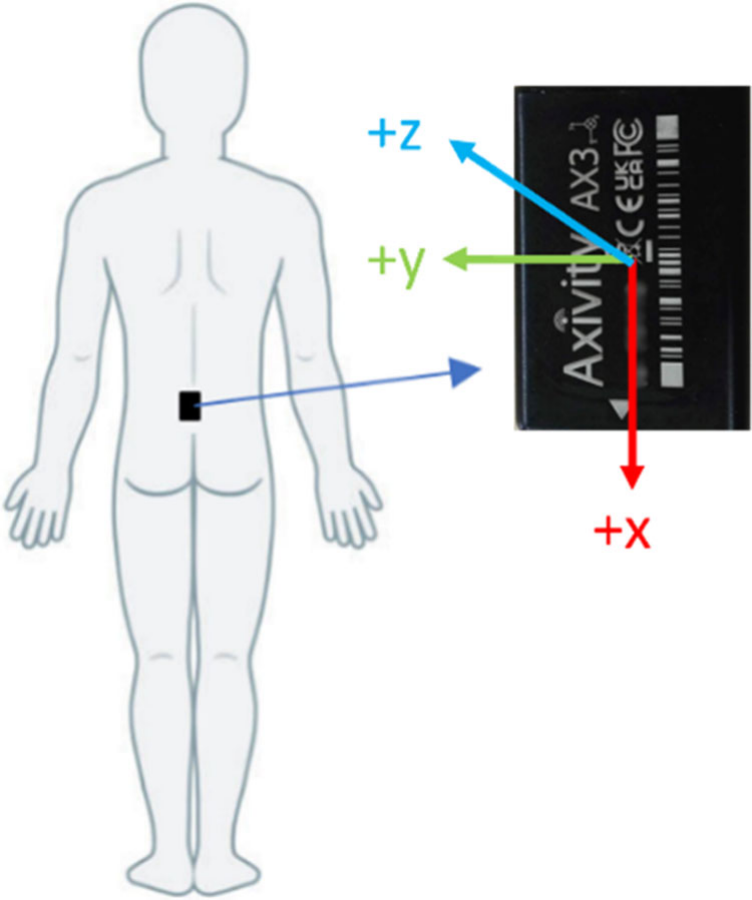
Placement of the accelerometer.

A back‐worn accelerometer (Axivity AX3; Axivity Ltd, UK) recording at a frequency of 100 Hz and a sensitivity range of ±8 g (g = gravitational acceleration) was positioned on the participants lower back (Figure 1) using an adhesive dressing (Tegaderm + Pad (3 M)). This was worn continuously including during showering and exercising during which time a bladder diary was also completed.

Accelerometery data was exported using Open Movement [Version 1.0.0.43] software. Nighttime voids were classified as periods of time when a participant moved from lying to standing and remained standing for a time period longer than the threshold value, determined from a sensitivity analysis. When standing, the gravitational force on an individual is 1 g in the vertical (x) direction. A datapoint was classified as standing if A_x_ ≥ 1 or ≤ −1. Positive and negative values were considered to account for incorrect accelerometer orientation. If the number of consecutive datapoints classified as standing was greater than or equal to the threshold value the standing period was deemed a nighttime void. For males that stand to pass urine, the threshold was 50, and for females or males that sit to pass urine, the threshold was 5.

Seven participants were recruited to the sub‐study (*n* = 5 male, *n* = 2 female). The mean age was 69, duration of PD 5 years and mean number of nocturia episodes over the diary period was 2.2. The detection of nocturia episodes per night are shown in Table 1. Due to the small sample size, statistical tests were not conducted.

**Table 1:** Nocturia episodes determined from the bladder‐diary and the accelerometer. Where Green = full concordance, Orange = A difference of one episode, Red = A difference of more than one episode.

Participant	Sex	Night 1		Night 2		Night 3		Mean	
		Bladder diary	Digital	Bladder diary	Digital	Bladder diary	Digital	Bladder diary	Digital
1	male	2	3	2	4	1	0	2.67	3.50
2	male	2	2	2	2	1	1	1.67	1.67
3	male	2	2	0	0	0	0	0.67	0.67
4	male	5	6	3	3	2	2	3.33	2.67
5	female	2	2	1	1	2	2	1.67	1.67
6	female	3	5	4	5	2	4	2.67	4.67
7	male	2	1	2	2	2	1	2.67	1.33

**Figure 2:**
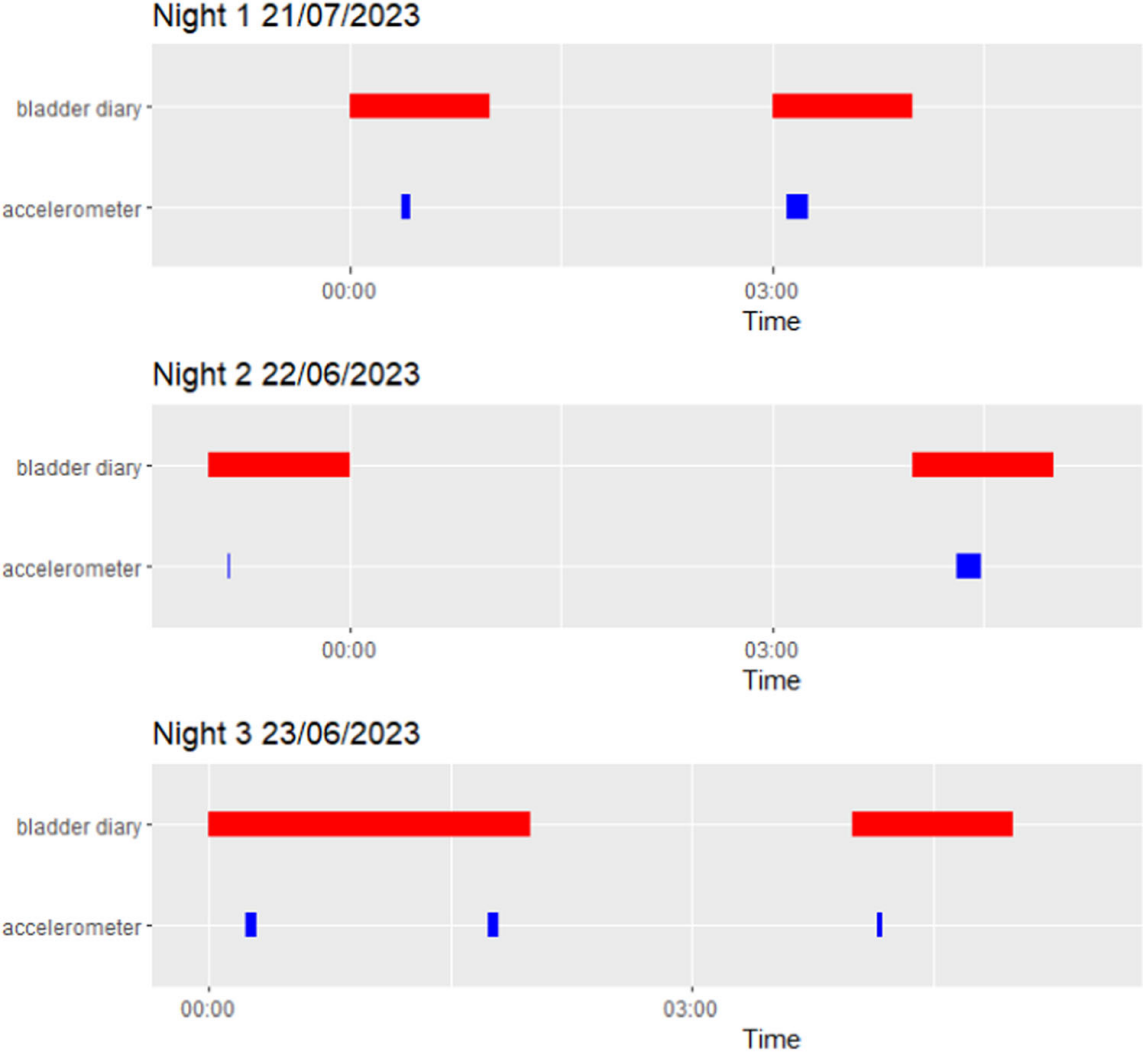
An example of nighttime voids detected from the diary and accelerometer.

The study suggested that accelerometry is a feasible method of capturing episodes of nocturia in Parkinson's disease. Participants were generally able to set the device up or utilize caregiver support and the majority had no issues during the testing period. Participant 1 reported the accelerometer become detached during the final night, hence captured no nocturia episodes. Agreement was shown between the diary and accelerometer with three participants having the same number of nocturia episodes per method. Furthermore, accelerometer detected voids provided greater temporal resolution than the diary where voids were recorded in 1‐h intervals, see Figure 2.

This study assumes any movement from lying to standing for a period of time is a void. However, as an individual may get up at night for other reasons, further refinement of the algorithm is required to only detect voids. Although a back‐worn accelerometer provides a stable signal not affected by tremor in PD, the potential of wrist‐worn accelerometers to detect voids should be explored as a more user‐friendly protocol. Finally, this study should be repeated on an adequately‐powered sample size to test the agreement between nocturia episodes detected with each method.

This study indicates the potential accelerometers have to provide an objective and less burdensome marker of nocturia in PD. Although the risk of detecting non‐urination events is present, significant differences may still be observed between baseline and post‐intervention assessments where nocturia episodes decrease. This work provides preliminary data that might serve to better capture urinary‐symptom related, outcome measures in clinical trials.

## Author Contributions

Conceptualization: Matthew D. Smith and Emily J. Henderson. Methodology: Anisha Cullen, Matthew D. Smith, and Emily J. Henderson. Formal analysis: Anisha Cullen. Resources: Matthew D. Smith and Emily J. Henderson. Data curation: Anisha Cullen. Writing–original draft preparation: Anisha Cullen, Matthew D. Smith, and Emily J. Henderson. Writing–review and editing: Anisha Cullen, Matthew D. Smith, and Emily J. Henderson. Visualization: Anisha Cullen. Supervision: Matthew D. Smith and E.J.H. Project administration: Anisha Cullen. Funding acquisition: Emily J. Henderson All authors have read and agreed to the published version of the manuscript.

## Ethics Statement

This study was conducted as part of an amendment to the STRIPE trial where ethical approval was granted by a UK Research Ethics Committee (REC) UK IRAS 295856.

## Consent

Written consent was obtained for all participants by members of the research team in alignment with Good Clinical Practice and the Mental Capacity Act 2019.

## Conflicts of Interest

The authors declare no conflicts of interest.

## Data Availability

Data may be made available on request to the Chief Investigator, Dr Emily Henderson.
